# Supplementary material for: Pyruvate Dehydrogenase Kinase 1 inhibition mediated oxidative phosphorylation enhancement in cartilage promotes osteoarthritis progression
Source: BMC Musculoskelet Disord. 2023 Jul 20;24:597. doi: 10.1186/s12891-023-06585-6 (PMC10357736; doi:10.1186/s12891-023-06585-6)
Supplement: Supplementary file 1 — Additional file 1. [file 12891_2023_6585_MOESM1_ESM.zip › MF.Gsea.1653627316242/GOMF_MODIFIED_AMINO_ACID_BINDING.html]

Details for gene set GOMF\_MODIFIED\_AMINO\_ACID\_BINDING[GSEA]

|  || Dataset | 55457\_collapsed\_to\_symbols.cls.cls #OA\_versus\_normal.cls.cls #OA\_versus\_normal\_repos |
| Phenotype | cls.cls#OA\_versus\_normal\_repos |
| Upregulated in class | OA |
| GeneSet | GOMF\_MODIFIED\_AMINO\_ACID\_BINDING |
| Enrichment Score (ES) | 0.2643643 |
| Normalized Enrichment Score (NES) | 1.1618882 |
| Nominal p-value | 0.18701299 |
| FDR q-value | 0.60285425 |
| FWER p-Value | 1.0 |
Table: GSEA Results Summary

  

Fig 1: Enrichment plot: GOMF\_MODIFIED\_AMINO\_ACID\_BINDING      
 Profile of the Running ES Score & Positions of GeneSet Members on the Rank Ordered List

  

| SYMBOL | TITLE | RANK IN GENE LIST | RANK METRIC SCORE | RUNNING ES | CORE ENRICHMENT || 1 | FOLR1 | folate receptor alpha [Source:HGNC Symbol;Acc:HGNC:3791] | 8 | 1.202 | 0.0526 | Yes |
| 2 | RPE65 | retinoid isomerohydrolase RPE65 [Source:HGNC Symbol;Acc:HGNC:10294] | 308 | 0.743 | 0.0622 | Yes |
| 3 | UROS | uroporphyrinogen III synthase [Source:HGNC Symbol;Acc:HGNC:12592] | 323 | 0.735 | 0.0937 | Yes |
| 4 | TREM2 | triggering receptor expressed on myeloid cells 2 [Source:HGNC Symbol;Acc:HGNC:17761] | 342 | 0.726 | 0.1244 | Yes |
| 5 | GSS | glutathione synthetase [Source:HGNC Symbol;Acc:HGNC:4624] | 502 | 0.658 | 0.1412 | Yes |
| 6 | APPL2 | "adaptor protein, phosphotyrosine interacting with PH domain and leucine zipper 2 [Source:HGNC Symbol;Acc:HGNC:18242]" | 586 | 0.628 | 0.1625 | Yes |
| 7 | MMACHC | metabolism of cobalamin associated C [Source:HGNC Symbol;Acc:HGNC:24525] | 642 | 0.608 | 0.1852 | Yes |
| 8 | ADGRB1 | adhesion G protein-coupled receptor B1 [Source:HGNC Symbol;Acc:HGNC:943] | 655 | 0.605 | 0.2110 | Yes |
| 9 | CD300A | CD300a molecule [Source:HGNC Symbol;Acc:HGNC:19319] | 730 | 0.587 | 0.2312 | Yes |
| 10 | GSTM3 | glutathione S-transferase mu 3 [Source:HGNC Symbol;Acc:HGNC:4635] | 1025 | 0.512 | 0.2310 | Yes |
| 11 | LANCL1 | LanC like 1 [Source:HGNC Symbol;Acc:HGNC:6508] | 1507 | 0.428 | 0.2124 | Yes |
| 12 | NOX4 | NADPH oxidase 4 [Source:HGNC Symbol;Acc:HGNC:7891] | 1563 | 0.418 | 0.2267 | Yes |
| 13 | RS1 | retinoschisin 1 [Source:HGNC Symbol;Acc:HGNC:10457] | 1682 | 0.401 | 0.2352 | Yes |
| 14 | GAS6 | growth arrest specific 6 [Source:HGNC Symbol;Acc:HGNC:4168] | 1942 | 0.368 | 0.2313 | Yes |
| 15 | GSDMD | gasdermin D [Source:HGNC Symbol;Acc:HGNC:25697] | 2121 | 0.347 | 0.2328 | Yes |
| 16 | MTHFS | methenyltetrahydrofolate synthetase [Source:HGNC Symbol;Acc:HGNC:7437] | 2155 | 0.343 | 0.2455 | Yes |
| 17 | CPS1 | carbamoyl-phosphate synthase 1 [Source:HGNC Symbol;Acc:HGNC:2323] | 2178 | 0.341 | 0.2588 | Yes |
| 18 | MGST2 | microsomal glutathione S-transferase 2 [Source:HGNC Symbol;Acc:HGNC:7063] | 2583 | 0.292 | 0.2403 | Yes |
| 19 | FOLR2 | folate receptor beta [Source:HGNC Symbol;Acc:HGNC:3793] | 2670 | 0.283 | 0.2461 | Yes |
| 20 | GPR143 | G protein-coupled receptor 143 [Source:HGNC Symbol;Acc:HGNC:20145] | 2730 | 0.277 | 0.2538 | Yes |
| 21 | FTCD | formimidoyltransferase cyclodeaminase [Source:HGNC Symbol;Acc:HGNC:3974] | 3064 | 0.244 | 0.2386 | Yes |
| 22 | SYT17 | synaptotagmin 17 [Source:HGNC Symbol;Acc:HGNC:24119] | 3084 | 0.241 | 0.2478 | Yes |
| 23 | GAP43 | growth associated protein 43 [Source:HGNC Symbol;Acc:HGNC:4140] | 3091 | 0.241 | 0.2580 | Yes |
| 24 | GSTM4 | glutathione S-transferase mu 4 [Source:HGNC Symbol;Acc:HGNC:4636] | 3144 | 0.235 | 0.2644 | Yes |
| 25 | MMUT | methylmalonyl-CoA mutase [Source:HGNC Symbol;Acc:HGNC:7526] | 3362 | 0.215 | 0.2570 | No |
| 26 | APPL1 | "adaptor protein, phosphotyrosine interacting with PH domain and leucine zipper 1 [Source:HGNC Symbol;Acc:HGNC:24035]" | 3532 | 0.199 | 0.2526 | No |
| 27 | SYT1 | synaptotagmin 1 [Source:HGNC Symbol;Acc:HGNC:11509] | 3829 | 0.173 | 0.2372 | No |
| 28 | SCARB1 | scavenger receptor class B member 1 [Source:HGNC Symbol;Acc:HGNC:1664] | 3979 | 0.158 | 0.2326 | No |
| 29 | SCARB2 | scavenger receptor class B member 2 [Source:HGNC Symbol;Acc:HGNC:1665] | 4048 | 0.152 | 0.2340 | No |
| 30 | TYMS | thymidylate synthetase [Source:HGNC Symbol;Acc:HGNC:12441] | 4328 | 0.126 | 0.2178 | No |
| 31 | MTHFR | methylenetetrahydrofolate reductase [Source:HGNC Symbol;Acc:HGNC:7436] | 4334 | 0.125 | 0.2230 | No |
| 32 | SYT13 | synaptotagmin 13 [Source:HGNC Symbol;Acc:HGNC:14962] | 4394 | 0.121 | 0.2238 | No |
| 33 | PTGES | prostaglandin E synthase [Source:HGNC Symbol;Acc:HGNC:9599] | 4399 | 0.121 | 0.2288 | No |
| 34 | CAVIN2 | caveolae associated protein 2 [Source:HGNC Symbol;Acc:HGNC:10690] | 4512 | 0.112 | 0.2250 | No |
| 35 | SYT5 | synaptotagmin 5 [Source:HGNC Symbol;Acc:HGNC:11513] | 4691 | 0.097 | 0.2155 | No |
| 36 | HAVCR1 | hepatitis A virus cellular receptor 1 [Source:HGNC Symbol;Acc:HGNC:17866] | 4902 | 0.080 | 0.2026 | No |
| 37 | SYT11 | synaptotagmin 11 [Source:HGNC Symbol;Acc:HGNC:19239] | 4969 | 0.075 | 0.2008 | No |
| 38 | JPH2 | junctophilin 2 [Source:HGNC Symbol;Acc:HGNC:14202] | 5061 | 0.068 | 0.1967 | No |
| 39 | CBS | cystathionine beta-synthase [Source:HGNC Symbol;Acc:HGNC:1550] | 5285 | 0.049 | 0.1815 | No |
| 40 | ANXA9 | annexin A9 [Source:HGNC Symbol;Acc:HGNC:547] | 5688 | 0.017 | 0.1509 | No |
| 41 | DHFR | dihydrofolate reductase [Source:HGNC Symbol;Acc:HGNC:2861] | 5805 | 0.008 | 0.1422 | No |
| 42 | OSBPL10 | oxysterol binding protein like 10 [Source:HGNC Symbol;Acc:HGNC:16395] | 5807 | 0.008 | 0.1425 | No |
| 43 | GSTM1 | glutathione S-transferase mu 1 [Source:HGNC Symbol;Acc:HGNC:4632] | 5848 | 0.005 | 0.1396 | No |
| 44 | PTGES2 | prostaglandin E synthase 2 [Source:HGNC Symbol;Acc:HGNC:17822] | 6269 | -0.025 | 0.1079 | No |
| 45 | SMPD3 | sphingomyelin phosphodiesterase 3 [Source:HGNC Symbol;Acc:HGNC:14240] | 6533 | -0.045 | 0.0894 | No |
| 46 | SYT12 | synaptotagmin 12 [Source:HGNC Symbol;Acc:HGNC:18381] | 6921 | -0.074 | 0.0625 | No |
| 47 | MFGE8 | milk fat globule EGF and factor V/VIII domain containing [Source:HGNC Symbol;Acc:HGNC:7036] | 7756 | -0.138 | 0.0036 | No |
| 48 | ANXA6 | annexin A6 [Source:HGNC Symbol;Acc:HGNC:544] | 8003 | -0.155 | -0.0087 | No |
| 49 | FOLR3 | folate receptor gamma [Source:HGNC Symbol;Acc:HGNC:3795] | 8329 | -0.180 | -0.0261 | No |
| 50 | GRAMD1B | GRAM domain containing 1B [Source:HGNC Symbol;Acc:HGNC:29214] | 8405 | -0.186 | -0.0237 | No |
| 51 | AXL | AXL receptor tyrosine kinase [Source:HGNC Symbol;Acc:HGNC:905] | 8565 | -0.199 | -0.0273 | No |
| 52 | CPNE1 | copine 1 [Source:HGNC Symbol;Acc:HGNC:2314] | 8642 | -0.204 | -0.0242 | No |
| 53 | ANXA2P2 | annexin A2 pseudogene 2 [Source:HGNC Symbol;Acc:HGNC:539] | 8911 | -0.225 | -0.0351 | No |
| 54 | PRMT3 | protein arginine methyltransferase 3 [Source:HGNC Symbol;Acc:HGNC:30163] | 9034 | -0.234 | -0.0342 | No |
| 55 | TLN1 | talin 1 [Source:HGNC Symbol;Acc:HGNC:11845] | 9131 | -0.242 | -0.0310 | No |
| 56 | HSPA8 | heat shock protein family A (Hsp70) member 8 [Source:HGNC Symbol;Acc:HGNC:5241] | 9140 | -0.243 | -0.0208 | No |
| 57 | GNMT | glycine N-methyltransferase [Source:HGNC Symbol;Acc:HGNC:4415] | 9208 | -0.248 | -0.0151 | No |
| 58 | MME | membrane metalloendopeptidase [Source:HGNC Symbol;Acc:HGNC:7154] | 9287 | -0.255 | -0.0099 | No |
| 59 | DPEP1 | dipeptidase 1 [Source:HGNC Symbol;Acc:HGNC:3002] | 9353 | -0.260 | -0.0034 | No |
| 60 | SYT2 | synaptotagmin 2 [Source:HGNC Symbol;Acc:HGNC:11510] | 9466 | -0.271 | -0.0002 | No |
| 61 | SCIN | scinderin [Source:HGNC Symbol;Acc:HGNC:21695] | 9742 | -0.296 | -0.0085 | No |
| 62 | SLC19A1 | solute carrier family 19 member 1 [Source:HGNC Symbol;Acc:HGNC:10937] | 9931 | -0.315 | -0.0092 | No |
| 63 | ASAP1 | "ArfGAP with SH3 domain, ankyrin repeat and PH domain 1 [Source:HGNC Symbol;Acc:HGNC:2720]" | 10276 | -0.349 | -0.0206 | No |
| 64 | SYTL2 | synaptotagmin like 2 [Source:HGNC Symbol;Acc:HGNC:15585] | 10681 | -0.391 | -0.0348 | No |
| 65 | MARK1 | microtubule affinity regulating kinase 1 [Source:HGNC Symbol;Acc:HGNC:6896] | 11031 | -0.434 | -0.0428 | No |
| 66 | OSBPL8 | oxysterol binding protein like 8 [Source:HGNC Symbol;Acc:HGNC:16396] | 11198 | -0.457 | -0.0355 | No |
| 67 | CPNE6 | copine 6 [Source:HGNC Symbol;Acc:HGNC:2319] | 11331 | -0.475 | -0.0248 | No |
| 68 | GSDMB | gasdermin B [Source:HGNC Symbol;Acc:HGNC:23690] | 11378 | -0.483 | -0.0070 | No |
| 69 | ANXA2 | annexin A2 [Source:HGNC Symbol;Acc:HGNC:537] | 11472 | -0.498 | 0.0078 | No |
| 70 | THBS1 | thrombospondin 1 [Source:HGNC Symbol;Acc:HGNC:11785] | 11498 | -0.501 | 0.0280 | No |
| 71 | ANXA13 | annexin A13 [Source:HGNC Symbol;Acc:HGNC:536] | 11806 | -0.551 | 0.0285 | No |
| 72 | HMGB1 | high mobility group box 1 [Source:HGNC Symbol;Acc:HGNC:4983] | 12170 | -0.636 | 0.0284 | No |
| 73 | FASN | fatty acid synthase [Source:HGNC Symbol;Acc:HGNC:3594] | 12194 | -0.643 | 0.0550 | No |
Table: GSEA details [plain text format]

  

Fig 2: GOMF\_MODIFIED\_AMINO\_ACID\_BINDING      
 Blue-Pink O' Gram in the Space of the Analyzed GeneSet

  

Fig 3: GOMF\_MODIFIED\_AMINO\_ACID\_BINDING: Random ES distribution      
 Gene set null distribution of ES for **GOMF\_MODIFIED\_AMINO\_ACID\_BINDING**
